# Exploring the ERP trace of task-set control in the composite design task-switching paradigm

**DOI:** 10.3389/fnhum.2025.1536926

**Published:** 2025-05-08

**Authors:** Fangyuan Zhou, Zhongjin Tian, Xiangqian Li

**Affiliations:** School of Psychology, Shanghai University of Sport, Shanghai, China

**Keywords:** task-set control, compound retrieval strategies, task-switching, Switch Positivity, N2, P3

## Abstract

**Introduction:**

In task-switching paradigms, Switch Positivity, the N2 difference wave, and the P3 difference wave are typically observed in the cue-target interval (CTI) design, where the cue precedes the target. The ERP components are indicative of task-set control processes (i.e., task-set reconfiguration and task-set inertia). However, in the composite design, where the cue and target appear simultaneously, these components are absent. Previous research has hypothesized that in the composite design task-switching experiments, participants may employ compound retrieval strategies based on associative learning to complete the tasks. This strategy circumvents task rules, thereby eliminating ERP components related to task-set control.

**Methods:**

This study aims to examine whether the use of compound retrieval strategies affects the task-set related ERP components. In Experiment 1, we manipulated participants’ semantic understanding of the target stimuli to control their strategies. Participants in the compound retrieval group exclusively used the compound retrieval strategy, while those in the control group could employ both the compound retrieval strategy and task rules. In Experiment 2, we varied the number of target stimuli to influence participants’ strategies, with participants in the task rule group utilizing task rules, and those in the control group permitted to use both task rules and the compound retrieval strategy.

**Results:**

The results revealed that Switch Positivity, the N2 difference wave, and the P3 difference wave were absent across all group conditions, regardless of the strategies employed.

**Discussion:**

These findings suggest that the disappearance of these ERP components in the composite design is not attributable to the use of compound retrieval strategies.

## Introduction

In task-switching paradigms, participants engage in two mixed cognitive tasks, either repeating a task (repeat trials) or switching between tasks (switch trials). Task-switching costs, defined as increased response times (RTs) and higher error rates (ERs) during switch trials compared to repeat trials, are well documented (Diamond, 2013; Kiesel et al., 2010; Koch et al., 2018). These costs arise from the additional cognitive control required during switch trials. Specifically, the *task-set reconfiguration account* posits that switch trials necessitate the reconfiguration of the previous task-set, a process not required in repeat trials, thereby inducing task-switching costs (Meiran, 1996; Meiran et al., 2000; Rogers and Monsell, 1995). In contrast, the *task-set inertia account* suggests that prior task-sets interfere with performance in switch trials while facilitating it in repeat trials, further contributing to task-switching costs (Allport et al., 1994; Allport and Wylie, 2000; Longman et al., 2014; Meiran et al., 2008). These accounts are not mutually exclusive, Vandierendonck et al. (2010) argued that task-switching costs result from the interplay of both processes. Collectively, these perspectives are referred to as the task-set control account.

### Cognitive control and ERP components

**Figure 1:**
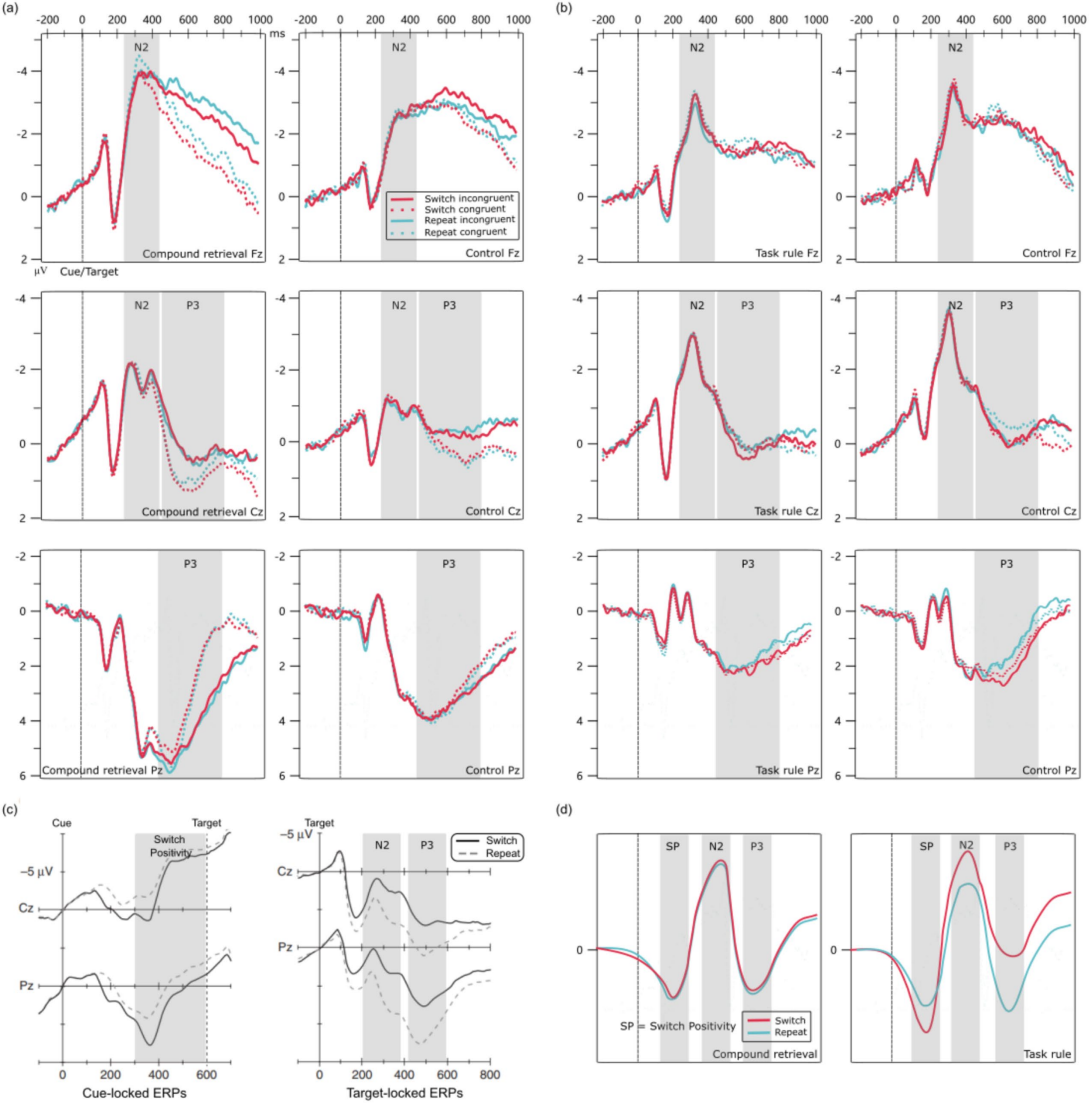
Grand average ERP waveforms. **(a)** ERP waveforms from Experiment 1; **(b)** ERP waveforms from Experiment 2; **(c)** Example of ERP waveforms from the task-switching paradigm using the CTI design (adapted from Karayanidis and Jamadar, 2014); **(d)** Hypothesized schematic for composite designs based on Karayanidis and Jamadar's (2014) account. The gray shading indicates the time window of the ERP component.

Event-related potential (ERP) studies have examined neural activity correlates of cognitive control in task-switching paradigms. Most ERP experiments employ cue-target intervals (CTIs), in which a task cue is predented first, followed by the target after a short delay, allowing for advance task preparation and enabling the differentiation of cue-locked from target-locked components (Figure 1c). During the cue-locked epoch, a positive deflection known as Switch Positivity occurs in centroparietal region around 400–600 ms post-cue, which is more pronounced in switch trials. During the target-locked epoch, switch trials exhibit larger frontocentral N2 and smaller centroparietal P3 components compared to repeat trials. Research indicates that Switch Positivity reflects task-set reconfiguration, while the N2 and P3 difference waves (i.e., switch - repeat) reflect task-set inertia (Barceló and Cooper, 2018; Cutini et al., 2021; Jamadar et al., 2010a; Gajewski et al., 2018; Karayanidis et al., 2011; Karayanidis and Jamadar, 2014; Kieffaber and Hetrick, 2005; Kray et al., 2020).

### Composite designs in ERP studies

Some ERP studies have adopted composite designs, where task cues and targets are presented simultaneously to prevent participants from preparing in advance (Swainson et al., 2006; Jamadar et al., 2010b). In such cases, the Switch Positivity is expected to emerge after the target onset, followed by the typical N2 and P3 components. However, in these experiments, the stimulus-locked Switch Positivity was not observed, even though significant behavioral task-switching costs were still evident. Furthermore, although the N2 and P3 components persisted, the amplitude differences between switch and repeat trials were minimal.

These morphological differences between ERP waveforms in composite and CTI designs can be explained by two approaches: either the ERP components related to task-switching no longer reflect task-set control process under composite design conditions, or CTI and composite designs suggest a qualitative distinction in processing between prepared and unprepared conditions.

Karayanidis and Jamadar (2014) adopted the latter approach and proposed that in composite designs, participants may utilize compound retrieval strategies based on associative learning instead of task rules (Forrest Charlotte et al., 2014; Li X. et al., 2019; Logan and Bundesen, 2004; Reisenauer and Dreisbach, 2013, 2014; Xu et al., 2021). Specifically, participants retrieve associations between cue-target compounds and responses, accessing the correct response from memory to complete tasks. We can examine the differences between the two approaches from a hierarchical task perspective (Kleinsorge and Heuer, 1999). The primary distinction between the compound retrieval strategy and task rules lies in how participants represent the stimulus–response mapping. Under task rules, participants extract different features from targets based on abstract categorization rules, ultimately using feature-response associations to produce the correct response (e.g., magnitude task: small → left). Since different tasks require distinct categorization rules, participants need cognitive control to maintain and switch task sets. In contrast, the compound retrieval strategy depends on direct stimulus–response associations, removing the need for task-set control and diminishing the distinction between switch and repeat trials.

The compound retrieval strategy minimizes differences between switch and repeat trials, thereby attenuating Switch Positivity and the N2 and P3 difference waves. Recent studies support that in composite designs, where advance task preparation is impossible, participants are more likely to employ compound retrieval strategies (Li X. et al., 2019; Li B. et al., 2019; Xu et al., 2021). Although Karayanidis and Jamadar did not empirically test this account, it remains theoretically plausible. Therefore, to determine whether Switch Positivity, N2, and P3 waveforms are influenced by compound retrieval strategies in composite design task-switching experiments, it is essential to systematically manipulate participants’ strategies.

### Interaction of task rules and compound retrieval strategies

Task rules and compound retrieval strategies are not mutually exclusive (Forrest Charlotte et al., 2014; Xu et al., 2021). In task-switching experiments with limited stimuli, some participants consistently employ compound retrieval strategies in certain trials. Similarly, in the studies by Swainson et al. (2006) and Jamadar et al. (2010b), participants cannot rely solely on compound retrieval strategies; otherwise, behavioral task-switching costs would disappear entirely (Forrest Charlotte et al., 2014; Li X. et al., 2019; Li B. et al., 2019; Reisenauer and Dreisbach, 2013, 2014). However, significant behavioral task-switching costs persist in these studies, indicating that the composite design merely increases the likelihood of employing compound retrieval strategies rather than eliminating the use of task rules.

### The manipulation of strategies

Strategies in task-switching experiments can be manipulated through two primary approaches. Li X. et al. (2019) utilized a mysterious symbol paradigm, where target stimuli consisted of symbols derived from Greek letters. In the compound retrieval group, participants were not informed of the symbols’ meanings, thus relying solely on associative compound retrieval strategies.^1^ In contrast, in the control group, participants were informed of the symbols’ meanings and conventional task rules, enabling the use of task rules. Nonetheless, due to limited task cues and targets, participants could also employ compound retrieval strategies. To further restrict compound retrieval strategies, Schneider (2018) coordinate switch paradigm can be applied. In the task rule group, target stimuli do not repeat during the experiment, reducing the feasibility of compound retrieval strategies. Additionally, in control groups with limited target stimuli, participants can apply both compound retrieval strategies and task rules. Thus, participants’ strategies can be manipulated across three conditions:

1. Compound Retrieval Group (Mysterious Symbol Paradigm): Participants rely solely on compound retrieval strategies.
2. Control Groups: Participants can utilize both compound retrieval strategies and task rules.
3. Task Rule Group (Coordinate Switch Paradigm): Participants rely solely on task rules.

### Response-congruency effects

In task-switching experiments, trials can also be categorized as congruent or incongruent. In congruent trials, the target elicits the same response across tasks, whereas in incongruent trials, the target requires different responses for each task. For example, under task rules (e.g., magnitude task: numbers smaller than five = left key, larger than five = right key; parity task: odd = left key, even = right key), the numeral “1” is classified as congruent, while the numeral “2” is classified as incongruent. Incongruent trials result in longer RTs and higher ERs due to response conflicts, known as response-congruency effects (Li B. et al., 2019; Meiran and Kessler, 2008; Monsell, 2003; Schneider, 2015; Schneider and Logan, 2015).

Response-congruency effects originate from two distinct sources. When participants use compound retrieval strategies, these effects are attributed to direct conflicts between the two target-response associations in incongruent trials. Conversely, when following task rules, participants need to categorize the targets along two dimensions (i.e., magnitude and parity). Hence, it is the feature-response associations in incongruent trials that lead to the response-congruency effects (Schneider, 2015). Effects mediated by task rules are generally smaller than those caused by direct conflicts (Li B. et al., 2019; Meiran and Kessler, 2008; Schneider, 2015).

### The present study

This study aimed to investigate the hypothesis that compound retrieval strategies may weaken the Switch Positivity, N2, and P3 difference waves between switch and repeat trials. In Experiment 1, we compared performance between participants in the compound retrieval group and the control group using a mysterious symbol paradigm. In Experiment 2, we compared performance between participants in the task rule group and the control group using a coordinate switch paradigm. We hypothesized that:

*H1*. In Experiment 1, participants in the compound retrieval group would exhibit smaller Switch Positivity, N2 and P3 difference waves compared to the control group.

*H2*. In Experiment 2, participants in the task rule group would demonstrate larger Switch Positivity, N2 and P3 difference waves compared to the control group.

Besides task-switching costs, another behavioral indicator distinguishing the compound retrieval strategy from task rules is the participants’ overall RT. When participants use the compound retrieval strategy, they can bypass task rules and directly retrieve strategies from long-term memory, which significantly reduces their RT (e.g., Forrest Charlotte et al., 2014; Li X. et al., 2019). Therefore, we hypothesized that:

*H3*. In Experiment 1, participants in the compound retrieval group would exhibit shorter RTs compared to the control group.

*H4*. In Experiment 2, participants in the control group would exhibit shorter RTs compared to the task rule group.

Additionally, response-congruency effects can also be used to distinguish between compound retrieval and task-rule strategies. Task-rule mediation would reduce response-congruency effects. Therefore, we hypothesized that:

*H5*. In Experiment 1, participants in the compound retrieval group would exhibit larger response-congruency effects compared to the control group.

*H6*. In Experiment 2, participants in the task rule group would demonstrate smaller response-congruency effects compared to the control group.

### Research ethics and open practice

This study adhered to the Declaration of Helsinki and was approved by the Ethics Committee of the authors’ university for Experiments 1 and 2. Additionally, behavioral data, EEG data, and analysis codes are available on the Open Science Framework at: https://osf.io/3b6rp/?view_only=6ff1f871749c4f7db4e7daea3b590e14.

## Experiment 1

### Materials and methods

#### Participants

Experiment 1 utilized a 2 (Participant Group: compound retrieval, control) × 2 (Trial Transition: switch, repeat) × 2 (Congruency: incongruent, congruent) mixed design, with participant group as the only between-subjects variable. G*Power (Faul et al., 2007) calculations indicated that 82 participants were required to detect a medium effect size (*f* = 0.25) with 80% power at *α* = 0.05. Although 90 participants from the authors’ university were initially recruited, eight were excluded due to excessive EEG noise, resulting in a final sample of 82 (41 per group; males = 34, mean age = 20.52, *SD* = 3.08). To be specific, participants were excluded if more than 40% of their trials contained artifacts exceeding ±120 μV, a threshold commonly used to identify significant noise in EEG data (Luck, 2014; Picton et al., 2000). The exclusion did not significantly affect group balance, and the remaining data were deemed suitable for robust statistical analysis. To ensure statistical power, we conducted real-time quality control. Each dataset was preprocessed immediately after collection, with replacement participants recruited when necessary due to excessive artifacts. Each participant received 80 RMB ($11) as compensation. All participants had normal or corrected-to-normal vision, were right-handed, and had no history of neurological or psychiatric disorders. Informed consent was obtained from participants prior to the experiment.

#### Mysterious symbol paradigm

In both experiments, participants were required to complete the training task and the experimental task. The training task was programmed by PsyToolkit software (Stoet, 2010, 2017), while the experimental task was programmed by Psychtoolbox software (Brainard, 1997; Kleiner et al., 2007) and presented on a 24-inch computer screen.

##### The training task

Participants in the two groups were assigned different training tasks. Participants in the compound retrieval group were not informed of the meanings of the symbol targets. Instead, they were instructed to memorize the response keys for eight different cue-target combinations (2 cues × 4 targets). The training involved two phases: memorization and testing. During the memorization phase, each trial displayed a cue-target combination at the center of the screen with corresponding key-press instructions (“A” or “L”) below, and participants were instructed to memorize the association between the response key and the cue-target combination without a time limit for responding. For example, when the combination “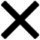 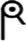
”was presented (“
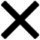
” above and “
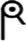
” below), participants would memorize and press the “L” key. This phase included 320 trials, with each combination appearing 40 times in a randomized order. The testing phase comprised 160 trials, presenting each combination 20 times, again randomized and ensuring that consecutive trials did not repeat the same cue-target compound (e.g., trials n and n-1 must have different cue-target compound). Trials were displayed with the cue-target compound at the center without key-press instructions, requiring participants to respond within 2,500 ms based on memory. Feedback was provided after each key press: 600 ms for correct responses and 2000 ms for incorrect or timed-out responses. Participants were required to achieve at least 85% accuracy to complete the training; those not meeting this threshold had to repeat the memorization phase.

**Figure 2:**
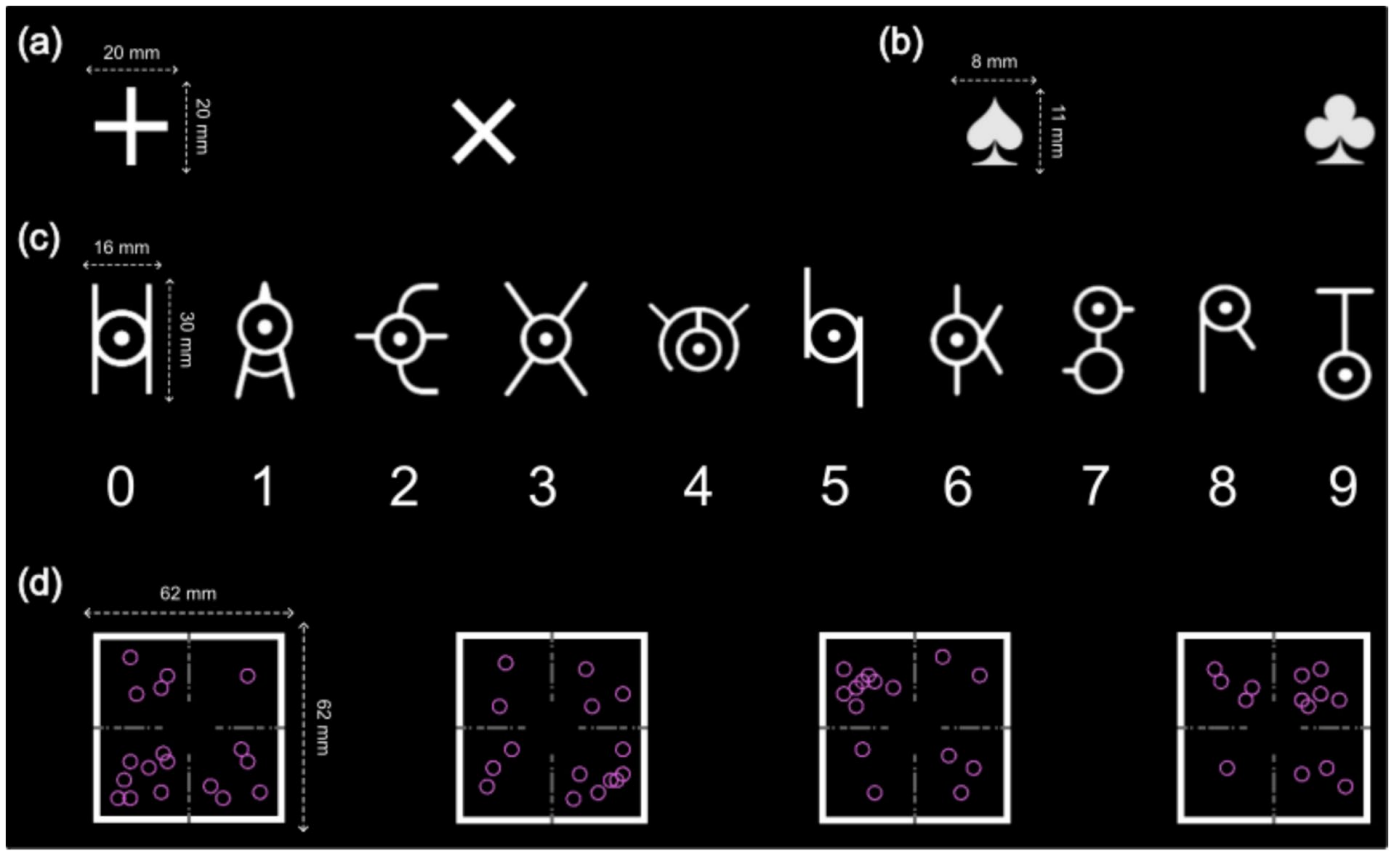
Cues and targets in Experiments 1 and 2. **(a)** Two cues in Experiment 1. “
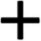
” represented the parity task, and “
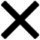
” represented the magnitude task; **(b)** Two cues in Experiment 2. “
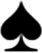
” represented the vertical task, and “
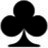
” represented the horizontal task; **(c)** The ten symbol targets and their corresponding numbers in Experiment 1. Additionally, the four symbol targets corresponding to 1, 2, 8, and 9 were used in the experimental task; **(d)** The four targets in the control group in Experiment 2.

Participants in the control group were required to memorize the meanings of ten symbol targets^2^ representing single digits (Figure 2c). The training started with a 400-trial memorization phase, each symbol appearing 40 times. During memorization, symbols were presented at the screen center with key-press instructions below, without a time limit. For example, when the symbol “
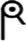
” appeared, participants were explicitly informed to press the number key “8.” After that, participants underwent the test phase. In Test Phase 1, participants had to press the corresponding number key from memory within 2,500 ms across 100 trials (each symbol appearing 10 times), receiving feedback for both correct (600 ms) and incorrect or timed-out responses (2,000 ms). Those achieving 90% accuracy advanced to Test Phase 2, involving 50 arithmetic tasks with symbols replacing numbers and requiring answers within 30,000 ms. For example, when the arithmetic task “
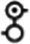
+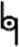
” appeared, participants had to press the number keys “1” and “2” to input the correct answer “12.” Participants needed 85% accuracy to complete this phase or otherwise returned to memorization.

##### The experimental task

Participants in both groups completed the same experimental task under different instructions. In the compound retrieval group, participants were asked to retrieve response keys for each cue-target combination from memory, while the control group selected responses based on parity and magnitude tasks. For the parity task, participants determined whether a number was odd (“A” key) or even (“L” key). For the magnitude task, they assessed whether a number was smaller than 5 (“A” key) or larger than 5 (“L” key). The experiment used two cues: one for parity and one for magnitude (Figure 2a), and four symbol targets.

**Figure 3:**
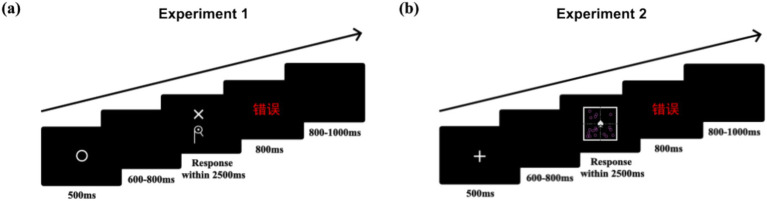
Schematic illustrations of the timeline of a trial: **(a)** Experiment 1, **(b)** Experiment 2.

Each trial began with a 500 ms fixation point (“⚪”),^3^ followed by a random blank screen for 600–800 ms. The cue (above) and target (below) then appeared simultaneously, and participants had 2,500 ms to respond by pressing “A” or “L.” Correct responses received no feedback; incorrect responses or timeouts were followed by 800 ms of feedback, then another random blank screen lasting 800–1,000 ms (Figure 3a). Participants completed one practice block (8 trials) and four formal blocks (80 trials each), totaling 320 trials. Additionally, the order of cues and targets was randomized, but we deliberately avoided consecutive repetitions of the same cue-target compound.

#### Procedure

Participants were randomly assigned to the compound retrieval or control group. The experiment took place over 2 days: on the first day, participants completed the training task online using their personal computers. After meeting the accuracy criterion, they visited the psychology lab on the second day, where they performed the experimental task in a soundproof room, wearing an EEG cap to record data.

#### EEG data recording and preprocessing

EEG data for Experiments 1 and 2 were recorded with a 64-channel Brain Products EEG system (bandwidth: DC-100 Hz; sampling rate: 1,000 Hz), using the international 10–20 system for electrode placement. The reference electrode was set to the FCz channel, and electrode impedance was maintained below 10 kΩ during recording. Preprocessing was conducted in MATLAB using the EEGLAB toolbox (Hediger et al., 2021). After applying channel locations, data were down-sampled to 250 Hz and band-pass filtered (0.1–30 Hz). Data segments were time-locked to 200 ms before and 1,000 ms after cue and target stimuli (−200 ms to 1,000 ms). Independent component analysis (ICA) was used to isolate ocular artifacts, such as blinks and eye movements. Subsequently, these artifact-related components were visually inspected and manually removed based on established criteria (Jung et al., 2000; Chaumon et al., 2015): (1) Spatial Distribution: strong activity over frontal electrodes (e.g., Fp1, Fp2, AF7, AF8); (2) Temporal Characteristics: high-amplitude, abrupt changes corresponding to blinks or saccades; and (3) Topographic Maps: dipolar patterns concentrated around the eyes. The artifact-related components were removed, and the remaining components were reconstructed to obtain artifact-free EEG data, a method validated for preserving neural signal integrity (Delorme et al., 2007; Klug and Gramann, 2021). Following this, baseline correction was applied using a baseline interval from −200 ms to 0 ms relative to the onset of the cue and target stimuli, which corresponds to the jittered 600–800 ms blank screen period used to establish the baseline. The data were then re-referenced using the average reference method, and trials with amplitudes exceeding ±120 μV were excluded. Finally, ERP waveforms for Experiment 1 (Figure 1a) and Experiment 2 (Figure 1b) were obtained by averaging all trials for each condition across participants.

#### Data analysis

Behavioral and EEG data from the experimental task were analyzed using R software (Version 4.3.1; R Core Team, 2023). RT and EEG analyses excluded the first trial of each block, error trials, and trials following errors.

Due to the absence of Switch Positivity (see results section), the analysis focused on N2 and P3 components. Based on prior studies (Chang et al., 2020; Gajewski et al., 2018; Zhou et al., 2020; Zhou and Qin, 2019), electrode selection for N2 included Fz and Cz, and for P3, Pz and Cz. The N2 time window was 240–420 ms, and the P3 time window was 420–800 ms post-cue and target onset. To reduce noise and enhance measurement reliability, we calculated ERP component amplitudes following established methods (Fan and Han, 2008; Luck and Gaspelin, 2017; Zhou et al., 2020; Zhou and Qin, 2019). Specifically, the peak of each component (e.g., the highest point for P3 or the lowest point for N2) was identified within its time window, and the amplitude was calculated as the average value across a 24-ms epoch (±12 ms) aligned with the peak.

We conducted 2 (Participant Group: compound retrieval, control) × 2 (Trial Transition: switch, repeat) × 2 (Congruency: incongruent, congruent) repeated measures ANOVAs for RT and ER, as well as 2 × 2 × 2 × 2 (Electrode: Fz and Cz for N2; Pz and Cz for P3) repeated measures ANOVAs for N2 and P3 amplitudes, with participant group as the only between-subjects variable. *Post hoc* tests using the Holm-Bonferroni correction (Holm, 1979) focused on task-switching costs and response-congruency effects.

To evaluate the evidence for null effects (Dienes, 2016), Bayesian analyses were conducted for N2 and P3 components with the BayesFactor package (Morey and Rouder, 2022). Default Cauchy priors (scale = 0.707) were specified, which are sensitive to medium-sized effects typical in cognitive neuroscience (Rouder et al., 2012; Wagenmakers et al., 2018).

For the N2 component, three Bayesian analyses were performed: (1) testing H₁ (amplitude difference between switch and repeat trials) against H₀ (no difference) within the compound retrieval group using Bayesian paired t-tests; (2) an identical comparison within the control group; and (3) testing H₁ (group difference in the amplitude of switch-repeat difference waves) against H₀ (no group difference) using Bayesian independent *t*-tests. Equivalent bayesian tests were applied to the P3 component. Bayes factors (BF₁₀) were interpreted using Kass and Raftery’s (1995) criteria: BF₁₀ > 3 indicates substantial evidence for H₁, BF₁₀ < 1/3 for H₀, and 1/3 < BF₁₀ < 3 as inconclusive.

### Results

#### Behavioral ANOVAs results

Two three-way repeated measures ANOVAs examined the effects of a between-subjects factor (participant group) and two within-subjects factors (trial transition, congruency) on RT and ER. The results are summarized in Table 1.

**Table 1:** Results of the RT and ER ANOVAs in Experiment 1, using participant group (compound retrieval, control) as a between-subjects factor, trial transition (switch, repeat) and congruency (incongruent, congruent) as within-subjects factors.

	RT				ER			
Effect	*F*	*df*	*p*	*η^2^_p_*	*F*	*df*	*p*	*η^2^_p_*
P	**102.63**	**1, 80**	**<0.001**	**0.56**	**51.03**	**1, 80**	**<0.001**	**0.39**
T	1.63	1, 80	0.205	0.02	**12.00**	**1, 80**	**0.001**	**0.13**
C	**221.70**	**1, 80**	**<0.001**	**0.73**	**127.94**	**1, 80**	**<0.001**	**0.62**
P × T	**11.89**	**1, 80**	**0.001**	**0.13**	3.27	1, 80	0.074	0.04
P × C	<0.01	1, 80	0.974	<0.01	**34.41**	**1, 80**	**<0.001**	**0.30**
T × C	0.88	1, 80	0.350	0.01	**4.99**	**1, 80**	**0.028**	**0.06**
P × T × C	**6.09**	**1, 80**	**0.016**	**0.07**	**5.49**	**1, 80**	**0.022**	**0.06**

P, participant group, T, trial transition, C, congruency, bold represents *p* < 0.05.

##### RT main effects

Significant main effects were found for participant group and congruency. RTs were longer in the control group than in the compound retrieval group (1081.16 ms vs. 768.23 ms) and for incongruent trials than for congruent trials (1043.86 ms vs. 805.53 ms).

##### RT task-switching related interactions

**Figure 4:**
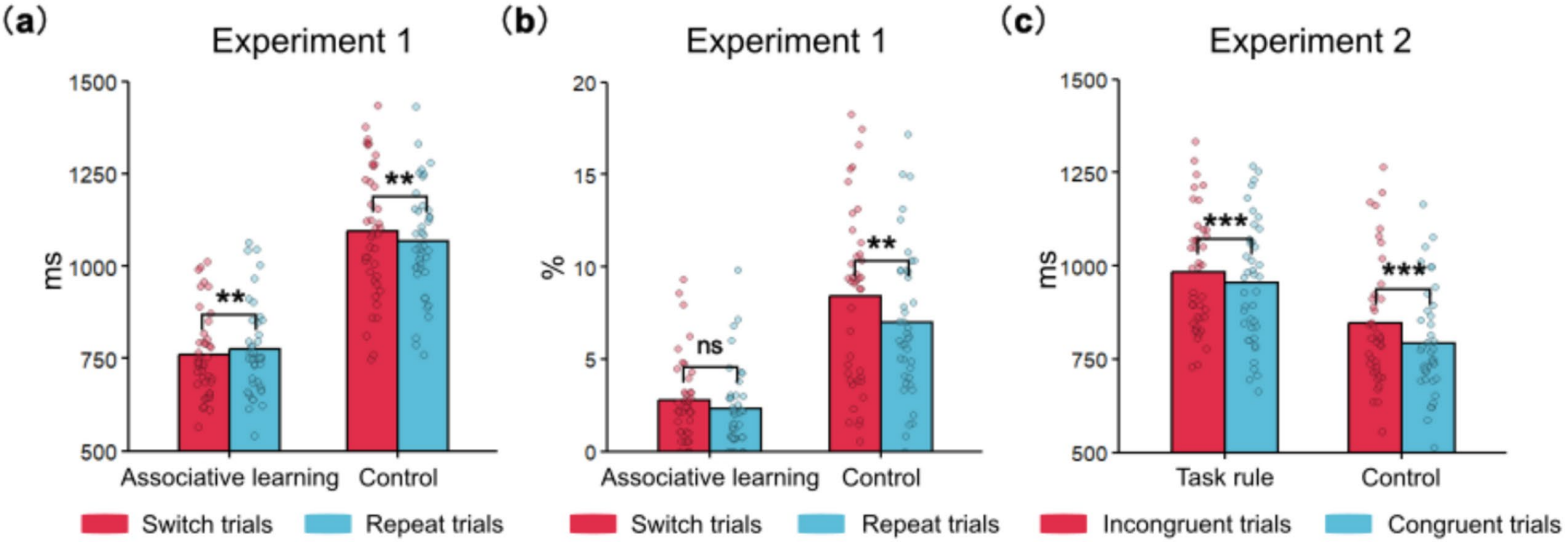
Main behavioral results of Experiments 1 and 2. **(a)** The interaction between participant group and trial transition on RT in Experiment 1; **(b)** The interaction between participant group and trial transition on ER in Experiment 1; **(c)** The interaction between participant group and congruency on RT in Experiment 2. Additionally, RT response-congruency effects in the task rule group were smaller than in the control group, *p* = 0.007. *** indicates *p* < 0.001, ** indicates *p* < 0.01, and * indicates *p* < 0.05. Each dot represents data from one participant.

The interaction between participant group and trial transition was significant (Figure 4a). Pairwise comparisons showed that in the control group, RTs for switch trials were longer than for repeat trials (1095.89 ms vs. 1066.43 ms), *p* = 0.002, *d* = 0.13. However, in the compound retrieval group, RTs for switch trials were shorter than for repeat trials (761.47 ms vs. 775.00 ms), *p* = 0.005, *d* = −0.07. In other words, task-switching costs were reversed.

The three-way interaction between participant group, trial transition, and congruency was significant. Further analysis showed that the interaction between trial transition and congruency was significant in the control group, *p* = 0.046, *η^2^_p_* = 0.10, but not in the compound retrieval group, *p* = 0.181, *η^2^_p_* = 0.04. Therefore, simple effects analyses were primarily performed in the control group. In incongruent trials of the control group, RTs for switch trials were longer than for repeat trials (1221.25 ms vs. 1178.88 ms), *p* = 0.009, *d* = 0.23. However, in congruent trials of the control group, there was no significant difference between switch and repeat trials (970.52 ms vs. 953.98 ms), *p* = 0.113.

##### ER main effects

Significant main effects were found for participant group, trial transition, and congruency. ERs were higher in the control group than in the compound retrieval group (7.68% vs. 2.52%), for switch trials than for repeat trials (5.56% vs. 4.64%), and for incongruent trials than for congruent trials (8.19% vs. 2.01%).

##### ER task-switching related interactions

The interaction between participant group and trial transition was marginally significant. To further explore the differences between two groups, post-hoc tests were conducted (Figure 4b). In the compound retrieval group, there was no significant difference between switch and repeat trials (2.74% vs. 2.30%), *p* = 0.088. However, in the control group, ERs for switch trials were higher than for repeat trials (8.38% vs. 6.99%), *p* = 0.002, *d* = 0.18.

##### ER congruency-related interactions

The interaction between participant group and congruency was significant. Post-hoc tests revealed that ERs were higher for incongruent trials than for congruent trials in the compound retrieval group (4.01% vs. 1.04%, *p* < 0.001, *d* = 0.97) and in the control group (12.37% vs. 2.99%, *p* < 0.001, *d* = 1.48). Additionally, response-congruency effects were smaller in the compound retrieval group than in the control group (2.97% vs. 9.38%), *p* < 0.001, *d* = −1.19.

The interaction between trial transition and congruency was significant. The three-way interaction between participant group, trial transition, and congruency was also significant. Further analysis revealed that the interaction between trial transition and congruency was significant in the control group, *p* = 0.012, *η^2^_p_* = 0.15, but not in the compound retrieval group, *p* = 0.914, *η^2^_p_* < 0.01. Therefore, simple effects analyses were primarily performed in the control group. In incongruent trials of the control group, the ERs for switch trials were higher than for repeat trials (13.60% vs. 11.15%), *p* = 0.002, *d* = 0.32. However, in congruent trials of the control group, there was no significant difference between switch and repeat trials (3.17% vs. 2.82%), *p* = 0.414.

#### ERP ANOVAs results

According to Karayanidis and Jamadar (2014), in a composite design, the Switch Positivity is expected to be elicited after target onset and followed by the typical post-target N2 and P3 components. However, we did not observe a significant Switch Positivity prior to the N2 component (Figure 1a). Therefore, we employed permutation analysis (Groppe et al., 2011; Maris and Oostenveld, 2007; Pernet et al., 2011) to further validate the ERP components preceding the N2 (0–240 ms). The results revealed no significant differences between switch and repeat trials in either the control group or the compound retrieval group across any electrodes (*ps* > 0.05). Thus, the Switch Positivity was not present in Experiment 1.

Therefore, only the N2 and P3 components were analyzed. Two four-way repeated measures ANOVAs examined the effects of a between-subjects factor (participant group) and three within-subjects factors (trial transition, congruency, electrode) on N2 and P3 amplitudes. The results are summarized in Table 2.

**Table 2:** Results of the N2 and P3 amplitude ANOVAs in Experiment 1, using participant group (compound retrieval, control) as a between-subjects factor, and trial transition (switch, repeat), congruency (incongruent, congruent), and electrode (Fz and Cz for N2; Pz and Cz for P3) as three within-subjects factors.

	N2				P3			
Effect	*F*	*df*	*p*	*η^2^_p_*	*F*	*df*	*p*	*η^2^_p_*
P	**6.89**	**1, 80**	**0.010**	**0.08**	**5.83**	**1, 80**	**0.018**	**0.07**
T	0.48	1, 80	0.492	0.01	1.83	1, 80	0.180	0.02
C	2.41	1, 80	0.124	0.03	**4.42**	**1, 80**	**0.039**	**0.05**
E	**48.50**	**1, 80**	**<0.001**	**0.38**	**117.39**	**1, 80**	**<0.001**	**0.59**
P × T	0.89	1, 80	0.347	0.01	1.04	1, 80	0.312	0.01
P × C	0.08	1, 80	0.780	<0.01	0.79	1, 80	0.377	0.01
P × E	0.83	1, 80	0.365	0.01	1.35	1, 80	0.248	0.02
T × C	0.98	1, 80	0.325	0.01	0.24	1, 80	0.629	<0.01
T × E	1.68	1, 80	0.198	0.02	**4.03**	**1, 80**	**0.048**	**0.05**
C × E	0.56	1, 80	0.456	0.01	**21.68**	**1, 80**	**<0.001**	**0.21**
P × T × C	0.11	1, 80	0.741	<0.01	0.71	1, 80	0.401	0.01
P × T × E	0.45	1, 80	0.504	0.01	1.60	1, 80	0.210	0.02
P × C × E	1.03	1, 80	0.313	0.01	0.28	1, 80	0.598	<0.01
T × C × E	2.58	1, 80	0.112	0.03	0.44	1, 80	0.507	0.01
P × T × C × E	0.19	1, 80	0.665	<0.01	1.52	1, 80	0.222	0.02

P, participant group, T, trial transition, C, congruency, E, electrode, bold represents *p* < 0.05.

##### N2 main effects

Significant main effects were found for participant group and electrode. The amplitude was smaller in the compound retrieval group than in the control group (−3.18 μV vs. −1.93 μV) and at Fz than at Cz (−3.40 μV vs. −1.71 μV).

##### P3 main effects

Significant main effects were found for participant group, congruency, and electrode. The amplitude was larger in the compound retrieval group than in the control group (3.18 μV vs. 2.10 μV), for congruent trials than for incongruent trials (2.79 μV vs. 2.49 μV), and at Pz than at Cz (4.76 μV vs. 0.52 μV).

##### P3 interactions

The interaction between trial transition and electrode was significant. Post-hoc tests revealed that at Pz, the amplitude was larger for repeat trials than for switch trials (4.89 μV vs. 4.63 μV), *p* = 0.021, *d* = 0.08. However, at Cz, there was no significant difference between repeat and switch trials (0.50 μV vs. 0.54 μV), *p* = 0.720.

The interaction between congruency and electrode was significant. Post-hoc tests revealed that at Cz, the amplitude was larger for congruent trials than for incongruent trials (0.89 μV vs. 0.15 μV), *p* < 0.001, *d* = 0.28. However, at Pz, there was no significant difference between congruent and incongruent trials (4.69 μV vs. 4.82 μV), *p* = 0.326.

#### Bayesian analyses results

##### N2

In the compound retrieval group, the Bayes factor (BF₁₀) for the amplitude difference between switch and repeat trials was 0.30, indicating that the data were 3.33 times more likely to be observed under H₀ than under H₁. In the control group, the BF₁₀ for the same comparison was 0.08, or 12.5 times more likely to be observed under H₀ than under H₁. When examining the group difference in the amplitude of switch-repeat difference waves, the BF₁₀ was 0.32, suggesting that the data were 3.13 times more likely to be observed under H₀ than under H₁.

##### P3

In the compound retrieval group, the Bayes factor (BF₁₀) for the amplitude difference between switch and repeat trials was 0.03, or 33.3 times more likely to be observed under H₀ than under H₁. In the control group, the BF₁₀ for the same comparison was 0.07, or 14.3 times more likely to be observed under H₀ than under H₁. When examining the group difference in the amplitude of switch-repeat difference waves, the BF₁₀ was 0.06, or 16.7 times more likely to be observed under H₀ than under H₁.

### Discussion

In Experiment 1, significant behavioral task-switching costs were observed only in the control group, not in the compound retrieval group. These results align with Li X. et al. (2019), suggesting that when participants cannot use task rules, they do not allocate additional cognitive resources to switch trials. Regarding ERP components, Switch Positivity was absent in both groups. Additionally, the ANOVA results indicated no significant difference in N2 and P3 amplitude between switch and repeat trials across participant groups. Furthermore, the Bayes factor provided substantial evidence (Kass and Raftery, 1995) in favor of the null hypothesis that participants had no N2 and P3 difference wave. These findings replicate those of previous studies (Jamadar et al., 2010b; Swainson et al., 2006) but contradict H1. Furthermore, participants’ RT in the compound retrieval group was 313 ms shorter than participants in the task rule group, which is consistent with previous studies (Forrest Charlotte et al., 2014; Li X. et al., 2019) and supports H3. However, contrary to H5, RT response-congruency effects showed no difference between the two groups, and ER effects were even smaller in the compound retrieval group.

Within the control group, task-switching costs were mainly observed in incongruent trials, likely because participants relied on task rules in these trials and favored the compound retrieval strategy in congruent trials. The compound retrieval strategy is straightforward in congruent trials, requiring only target-response association memorization. In incongruent trials, it involves biconditional discrimination, which is more cognitively demanding (Harris and Livesey, 2008; Li X. et al., 2019; Livesey et al., 2011).

Task-switching costs were reversed in the compound retrieval group, though the effect size was small (*d* = −0.07). In Experiment 1, cue-target compounds never repeated across consecutive trials; thus, target stimuli repeated only in switch trials (as task cues already repeated in repeat trials). Using a compound retrieval strategy, participants could bypass task cue processing in congruent trials. As a result, consecutive identical congruent targets might provide a slight RT advantage in switch trials.

## Experiment 2

### Materials and methods

#### Participants

Experiment 2 followed a design similar to Experiment 1. G*Power indicated a sample size of 82 participants (41 per group) for medium power (0.8), effect size *f* = 0.25, and *α* = 0.05. A total of 85 naive participants were recruited from the authors’ university, with three excluded due to excessive EEG noise. The same exclusion criteria as in Experiment 1 were applied, with no significant impact on group balance. Ultimately, 82 participants (41 per group; 23 males, mean age = 20.66, *SD* = 2.28) completed the experiment. Each received 80 RMB as compensation.

#### Coordinate switch paradigm

The coordinate switch paradigm required participants to count hollow circles on a coordinate system while alternating between a vertical task (determining whether the top or bottom half had more circles; top = “A” key, bottom = “L” key) and a horizontal task (determining whether the left or right half had more circles; left = “A” key, right = “L” key). In Experiment 2, a spade symbol indicated the vertical task, and a club symbol indicated the horizontal task (Figure 2b).

The primary difference between the task rule and control groups was target variability. In the task rule group, the position and number of hollow circles were randomized each trial, subject to four constraints: (1) circles could not overlap with the task cue or coordinate axes; (2) each quadrant contained 1 to 9 circles; (3) the number of circles in the top (quadrants 1 and 2) and bottom (quadrants 3 and 4) halves were unequal; and (4) the number of circles in the left (quadrants 2 and 3) and right (quadrants 1 and 4) halves were also unequal. Each circle had approximately 10,000 potential positions, making target replication nearly impossible. In contrast, the control group used only four distinct targets (Figure 2d).

In each trial, a fixation point (“
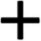
”) first appeared for 500 ms, followed by a random blank screen (600–800 ms). Then, the cue and target appeared simultaneously, requiring a response within 2,500 ms. Correct responses received no feedback; incorrect responses or timeouts were followed by 800 ms of feedback, then another random blank screen lasting 800–1,000 ms (Figure 3b). Additionally, the order of cues and targets was randomized, but we deliberately avoided consecutive repetitions of the same cue-target compound.

#### Procedure

The experiment took place over 2 days. On the first day, participants completed an online training session for the coordinate switch paradigm, consisting of six blocks with 72 trials each (432 trials total). Participants achieving 85% accuracy or higher advanced to the next phase; those who did not meet the criterion repeated the training until they succeeded. On the second day, participants visited the laboratory for the formal EEG experiment, completing one practice block (8 trials) and four formal blocks (80 trials each, totaling 320 trials). Only data from the EEG experiment were analyzed.

#### Data analysis

In the EEG data analysis, since Switch Positivity was not observed, the focus shifted to two ERP components: N2 and P3. Based on the waveforms (Figure 1b), the N2 time window was 260–360 ms, and the P3 time window was 460–780 ms after cue and target onset. All other aspects were consistent with Experiment 1.

### Results

#### Behavioral ANOVAs results

Two three-way repeated measures ANOVAs examined the effects of a between-subjects factor (participant group) and two within-subjects factors (trial transition, congruency) on RT and ER. The results are summarized in Table 3.

**Table 3:** Results of the RT and ER ANOVAs in Experiment 2, using participant group (task rule, control) as a between-subjects factor, trial transition (switch, repeat) and congruency (incongruent, congruent) as within-subjects factors.

	RT				ER			
Effect	*F*	*df*	*p*	*η^2^_p_*	*F*	*df*	*p*	*η^2^_p_*
P	**18.01**	**1, 80**	**<0.001**	**0.18**	1.24	1, 80	0.269	0.02
T	**42.68**	**1, 80**	**<0.001**	**0.35**	3.75	1, 80	0.056	0.04
C	**69.24**	**1, 80**	**<0.001**	**0.46**	**85.30**	**1, 80**	**<0.001**	**0.52**
P × T	1.50	1, 80	0.224	0.02	0.69	1, 80	0.407	0.01
P × C	**5.51**	**1, 80**	**0.021**	**0.06**	1.56	1, 80	0.216	0.02
T × C	0.43	1, 80	0.514	0.01	1.90	1, 80	0.172	0.02
P × T × C	0.33	1, 80	0.569	<0.01	1.18	1, 80	0.280	0.01

P, participant group; T, trial transition; C, congruency, bold represents *p* < 0.05.

##### RT main effects

Significant main effects were found for participant group, trial transition, and congruency. RTs were longer in the task rule group than in the control group (968.78 ms vs. 820.29 ms), for switch trials compared to repeat trials (911.85 ms vs. 877.22 ms), and for incongruent trials compared to congruent trials (915.93 ms vs. 873.14 ms).

##### RT congruency-related interactions

A significant interaction between participant group and congruency was observed (Figure 4c). *Post hoc* tests revealed that both the task rule group (984.14 ms vs. 953.42 ms, *p* < 0.001, *d* = 0.19) and the control group (847.72 ms vs. 792.86 ms, *p* < 0.001, *d* = 0.33) had longer RTs for incongruent compared to congruent trials. Additionally, response-congruency effects were smaller in the task rule group than in the control group (30.72 ms vs. 54.86 ms), *p* = 0.007, *d* = −0.43.

##### ER main effects

The main effect of congruency was significant; ERs for incongruent trials were higher than for congruent trials (5.79% vs. 1.06%).

#### ERP ANOVAs results

Based on visual inspection (Figure 1b), no Switch Positivity was observed. Furthermore, permutation analysis revealed no significant differences between switch and repeat trials prior to the N2 component (0–260 ms) in either the task rule group or the control group across any electrodes (*ps* > 0.05). Therefore, Switch Positivity was not present in Experiment 2.

Therefore, only the N2 and P3 components were analyzed. Two four-way repeated measures ANOVAs examined the effects of a between-subjects factor (participant group) and three within-subjects factors (trial transition, congruency, electrode) on N2 and P3 amplitudes. The results are summarized in Table 4.

**Table 4:** Results of the N2 and P3 amplitude ANOVAs in Experiment 2, using participant group (compound retrieval, control) as a between-subjects factor, and trial transition (switch, repeat), congruency (incongruent, congruent), and electrode (Fz and Cz for N2; Pz and Cz for P3) as three within-subjects factors.

	N2				P3			
Effect	*F*	*df*	*p*	*η^2^_p_*	*F*	*df*	*p*	*η^2^_p_*
P	0.98	1, 80	0.326	0.01	0.01	1, 80	0.923	<0.01
T	0.12	1, 80	0.726	<0.01	3.28	1, 80	0.074	0.04
C	1.90	1, 80	0.172	0.02	1.45	1, 80	0.232	0.02
E	<0.01	1, 80	0.948	<0.01	**58.87**	**1, 80**	**<0.001**	**0.42**
P × T	0.03	1, 80	0.872	<0.01	0.19	1, 80	0.661	<0.01
P × C	0.01	1, 80	0.916	<0.01	0.73	1, 80	0.396	0.01
P × E	0.36	1, 80	0.548	<0.01	0.94	1, 80	0.336	0.01
T × C	0.24	1, 80	0.624	<0.01	0.32	1, 80	0.574	<0.01
T × E	1.66	1, 80	0.201	0.02	0.07	1, 80	0.791	<0.01
C × E	1.29	1, 80	0.259	0.02	0.54	1, 80	0.464	0.01
P × T × C	0.32	1, 80	0.574	<0.01	0.25	1, 80	0.616	<0.01
P × T × E	0.11	1, 80	0.745	<0.01	0.03	1, 80	0.861	<0.01
P × C × E	0.01	1, 80	0.922	<0.01	0.05	1, 80	0.818	<0.01
T × C × E	1.42	1, 80	0.237	0.02	0.82	1, 80	0.368	0.01
P × T × C × E	1.13	1, 80	0.290	0.01	2.76	1, 80	0.101	0.03

P, participant group; T, trial transition; C, congruency; E, electrode, bold represents *p* < 0.05.

##### P3 main effects

The main effect of electrode was significant; the amplitude at Pz was larger than at Cz (2.34 μV vs. 0.08 μV).

#### Bayesian analyses results

##### N2

In the task rule group, the Bayes factor (BF₁₀) for the amplitude difference between switch and repeat trials was 0.07, indicating that the data were 14.3 times more likely to be observed under H₀ than under H₁. In the control group, the BF₁₀ for the same comparison was 0.08 indicating that the data were 12.5 times more likely to be observed under H₀ than under H₁. When examining the group difference in the amplitude of switch-repeat difference waves, the BF₁₀ was 0.11, indicating that the data were 9.1 times more likely to be observed under H₀ than under H₁.

##### P3

In the task rule group, the Bayes factor (BF₁₀) for the amplitude difference between switch and repeat trials was 0.26, indicating that the data were 3.8 times more likely to be observed under H₀ than under H₁. In the control group, the BF₁₀ for the same comparison was 0.52, indicating that the data were 1.92 times more likely to be observed under H₀ than under H₁. When examining the group difference in the amplitude of switch-repeat difference waves, the BF₁₀ was 0.09, indicating that the data were 11.1 times more likely to be observed under H₀ than under H₁.

### Discussion

In Experiment 2, task-switching costs did not differ significantly between the task rule and control groups. Regarding ERP components, Switch Positivity was absent in both groups, and there were no significant differences in N2 and P3 amplitudes between switch and repeat trials. In addition, the Bayes factor provided substantial evidence (Kass and Raftery, 1995) in favor of the null hypothesis that participants had no N2 and P3 difference wave. These findings replicate those of Experiment 1 and previous studies (Jamadar et al., 2010b; Swainson et al., 2006) but contradict H2. Nevertheless, we found that the RT of participants in the control group was 148 ms shorter than that of the task rule group, which supports H4, indicating that participants in the control group indeed used the compound retrieval strategy in some trials. Additionally, RT response-congruency effects were smaller in the task rule group than in the control group, consistent with H6.

## General discussion

This study aimed to determine whether Switch Positivity, the N2 difference wave, and the P3 difference wave in a composite design task-switching experiment are influenced by the compound retrieval strategy. In Experiment 1, we compared the performance of the compound retrieval group (participants who only used the compound retrieval strategy) with the control group (participants who used both task rules and the compound retrieval strategy). In Experiment 2, we compared the task rule group (participants who were unable to use the compound retrieval strategy) with the control group. However, the results revealed that Switch Positivity was absent across all groups. Although N2 and P3 components were observed, their difference waves (switch vs. repeat trials) were not significant. These findings replicate those of previous studies (Swainson et al., 2006; Jamadar et al., 2010b) and challenge the hypothesis proposed by Karayanidis and Jamadar (2014). Specifically, our results indicate that the compound retrieval strategy does not affect these ERP components in composite design task-switching experiments.

In the CTI design, Switch Positivity reflects task-set reconfiguration, while the N2 and P3 difference waves may reflect task-set inertia (Jamadar et al., 2010a; Karayanidis and Jamadar, 2014). Thus, the absence of these ERP components in the composite design could be due to weakened task-set control because participants applied alternative strategies that require less task-set control (as proposed by Karayanidis and Jamadar, 2014), or the ERP components may no longer reflect these processes despite normal task-set control. Our results do not support the first explanation. Therefore, we must consider why ERP components fail to reflect task-set control processes in the composite design.

One possibility is that when cues and targets are presented simultaneously, participants process them in parallel rather than first processing the cue and then the target, as proposed by Karayanidis and Jamadar (2014). This parallel processing could lead to temporal overlap between ERP components, making it difficult to observe waveforms in the composite condition that are morphologically similar to those in the CTI condition. Specifically, Switch Positivity shares a similar time window (400–600 ms post-stimulus) with the P3 component. Consequently, the P3 elicited by the target might mask or merge with the Switch Positivity, rendering it undetectable. Similarly, while the N2 and P3 in the CTI design typically reflect the task-set inertia process; however, in the composite design, they might simultaneously reflect both the task-set reconfiguration and task-set inertia processes. The neural competition between these two processes could obscure the differences between switch and repeat trials.

Additionally, our participants were young university students with peak executive functions. Consequently, their N2 and P3 difference waves were inherently small—stronger executive functions lead to more effective resolution of task-set interference, thereby reducing N2 and P3 difference waves (Karayanidis et al., 2011; Chang et al., 2020).

This is to say, in the present study, the composite design is not ideally suited for detecting ERP components related to task-set control, and our participants may not exhibit salient N2 and P3 difference waves. Therefore, it is not surprising that N2 and P3 difference waves were not observed in the present study. Future studies should compare individuals with varying cognitive abilities to better understand how task-set inertia modulates ERP components in composite designs.

In short, we propose that the absence of these ERP components does not necessarily imply the lack of task-set control processes but instead suggests that the task-switching experiment with composite design may not be well-suited for observing these ERP components related to task-set control. Future studies are needed to further investigate and validate this possibility.

### Strategy manipulation and behavioral results

Some behavioral results may have been influenced by participants’ strategies. In Experiment 1, the compound retrieval group demonstrated shorter RTs than the control group. Similarly, in Experiment 2, the control group showed shorter RTs than the task rule group. This pattern likely reflects the use of the compound retrieval strategy, where participants achieve shorter RTs through direct response retrieval without relying on task rules (Forrest Charlotte et al., 2014; Li X. et al., 2019; Li B. et al., 2019).

Moreover, task-switching costs typically disappear when participants fully adopt a compound retrieval strategy (Li X. et al., 2019; Li B. et al., 2019; Reisenauer and Dreisbach, 2013, 2014). In Experiment 1, the absence of task-switching costs in the compound retrieval group was consistent with previous findings. However, in Experiment 2, both the control and task rule groups showed significant and comparable task-switching costs. This suggests that, although participants in the control group could use both compound retrieval and task rules, their task-switching costs were similar to those of the task rule group, which relied solely on task rules. One interpretation is that control group participants may have intermittently employed both task rules and compound retrieval strategies. In such cases, task-switching costs might arise from different mechanisms, as some studies suggest that “non-cognitive control” factors contribute to these costs (Logan and Bundesen, 2003; Schmidt et al., 2020; Forrest Charlotte et al., 2014). For example, Forrest Charlotte et al. (2014) proposed that associative learning networks could generate task-switching costs even with compound retrieval strategies. Thus, the similar task-switching costs observed in the task rule and control groups may reflect different underlying processes—task-set control combined with associative learning networks in the control group, and pure task-set control in the task rule group. However, this interpretation requires further investigation.

From another perspective, the behavioral task-switching costs in Experiment 2 did not effectively reflect the strategies used by participants. Our results suggested that to avoid the influence of “non-cognitive control” factors—especially associative learning networks—on task-switching costs and to obtain a clearer measure of task-set control, utilizing a task-switching paradigm where target stimuli do not repeat is a suitable approach (Schneider, 2015, 2018).

Lastly, in Experiment 2, response-congruency effects were smaller in the task rule group than in the control group. This difference may be due to some control group participants using the compound retrieval strategy. Previous studies (Li B. et al., 2019; Meiran and Kessler, 2008; Schneider, 2015) suggest that response-congruency effects are smaller when task rules are used compared to compound retrieval strategies. However, in Experiment 1, RT response-congruency effects showed no difference between the groups, and ER effects were even smaller in the compound retrieval group. This finding suggests that compound retrieval may not be the sole factor influencing response-congruency effects. We speculate that this might be due to the similarity between the training and experimental tasks in Experiment 1: the compound retrieval group’s training and experimental tasks involved remembering and recalling cue-target compounds, while control group participants memorized symbols during training but performed conventional task-switching during the experiment. As a result, the control group may have been less familiar with the task, potentially reducing RTs and ERs, particularly in more challenging incongruent trials.

Notably, the observed behavioral differences between different strategy groups, while consistent with strategic modulation, warrant careful consideration of alternative interpretations. For example, RT variations bewteen participant groups could also reflect differences in stimulus familiarity. In Experiment 2, the task rule group encountered non-repeating stimuli, which might reduce familiarity and potentially prolong RTs. Similarly, the magnitude of response-congruency effects may be influenced by stimulus set size. Specifically, in the task rule group, non-repeating stimuli could introduce random trial-to-trial variability, increasing system noise and obscuring the measurement of congruency effects arising from conflicts between target dimensions.

### Theoretical contribution

In terms of ERP components, we propose that in the composite design, participants may process cues and targets in parallel, leading to Switch Positivity failing to reflect task-set reconfiguration and N2/P3 difference waves failing to reflect task-set inertia. However, inline with previous studies, task-switching costs were significant in both of our experiments. Thus, in the composite design, task-switching costs may provide a more reliable measure of cognitive control compared to ERP components. These implications highlight the importance of carefully considering experimental design when interpreting cognitive control processes with ERP results.

## Conclusion

1. Compound retrieval strategies do not account for the disappearance of ERP components (Switch Positivity, N2 difference wave, P3 difference wave) in composite design task-switching experiments.
2. Behavioral task-switching costs more accurately reflect task-set control than ERP components in composite designs.

## Data Availability

The datasets presented in this study can be found in online repositories. The names of the repository/repositories and accession number(s) can be found at: https://osf.io/3b6rp/?view_only=2d36800c40b04d228a83fea0e1aa37a8.

## Footnotes

^1^As Li X. et al. (2019) noted, human participants typically do not fully revert to associative learning. When stating that participants rely on compound retrieval strategies, it implies that, compared to repeat trials, participants generally do not exert additional cognitive control in switch trials, as the same set of rules applies to both trial types.

^2^Notably, although only four symbol targets were used in the experimental task, participants were required to memorize numbers for ten symbol targets during training. This was implemented to prevent participants from easily recognizing that they could bypass task rules and instead use compound retrieval strategies in the experimental task.

^3^In most ERP studies, the “**+**” symbol is typically employed as the fixation point, as illustrated in Experiment 2. However, in Experiment 1, the “**⚪**” was used as the fixation point because the “**+**” already served as the cue for the parity task.
